# New Books

**Published:** 2010-03

**Bottled and Sold: The Story Behind Our Obsession with Bottled Water**

Peter H. Gleick

Washington, DC:Island Press, 2010. 288 pp. ISBN: 978-1-59726-528-7, $26.95

**Climate and Society: Climate as Resource, Climate as Risk**

Nico Stehr, Hans von Storch

Hackensack, NJ:World Scientific, 2009. 152 pp. ISBN: 978-981-4280-53-2, $60

**Crucial Issues in Climate Change and the Kyoto Protocol: Asia and the World**

Kheng-Lian Koh, Lin Heng Lye, Jolene Lin, eds.

Hackensack, NJ:World Scientific, 2009. 596 pp. ISBN: 978-981-4277-52-5, $98

**Energy, The Environment and Climate Change**

Peter E. Hodgson

Hackensack, NJ:World Scientific, 2010. 200 pp. ISBN: 978-1-84816-415-4, $78

**Environmental Social Science: Human–Environment Interactions and Sustainability**

Emilio Moran

Hoboken, NJ:John Wiley & Sons, 2010. 232 pp. ISBN: 978-1-4051-0573-6, $89.95

**Expanding Biofuel Production: Sustainability and the Transition to Advanced Biofuels**

Patricia Koshel, Kathleen McAllister

Washington, DC:National Academies Press, 2010. 194 pp. ISBN: 978-0-309-14714-9, $43.50

**Hidden Costs of Energy: Unpriced Consequences of Energy Production and Use**

Committee on Health, Environmental, and Other External Costs and Benefits of Energy Production and Consumption; National Research Council

Washington, DC:National Academies Press, 2010. 350 pp. ISBN: 978-0-309-14636-4, $54

**Historical Energy Statistics: Global, Regional and National Trends Since Industrialization**

T.S. Gopi Rethinaraj, Clifford E. Singer

Hackensack, NJ:World Scientific, 2010. 400 pp. ISBN: 978-981-270-739-0, $118

**Internationalization of the Nuclear Fuel Cycle: Goals, Strategies, and Challenges**

U.S. Committee on the Internationalization of the Civilian Nuclear Fuel Cycle; Committee on International Security and Arms Control; National Academy of Sciences and National Research Council

Washington, DC:National Academies Press, 2009. 160 pp. ISBN: 978-0-309-12660-1, $45.75

**Linkages of Sustainability**

Thomas E. Graedel, Ester van der Voet, eds.

Cambridge, MA:MIT Press, 2009. 430 pp. ISBN: 978-0-262-01358-1, $40

**Progress in Bioethics: Science, Policy, and Politics**

Jonathan D. Moreno, Sam Berger, eds.

Cambridge, MA:MIT Press, 2010. 308 pp. ISBN: 978-0-262-13488-0, $29

**Statistical Bioinformatics: For Biomedical and Life Science Researchers**

Jae K. Lee

Hoboken, NJ:John Wiley & Sons, 2010. 384 pp. ISBN: 978-0-471-69272-0, $99.95

**The Implications of China’s Rising Energy Use**

Peter Sheehan

Hackensack, NJ:World Scientific, 2010. 200 pp. ISBN: 978-981-279-406-2, $69

**The World According to Monsanto: Pollution, Corruption, and the Control of Our Food Supply**

Marie-Monique Robin

New York:New Press, 2009. 384 pp. ISBN: 978-1-59558-426-7, $26.95

**Water Security**

World Economic Forum Water Initiative

Washington, DC:Island Press, 2010. 300 pp. ISBN: 978-1-59726-735-9, $60

